# Parental Phubbing and Preschoolers’ Eating Problems: The Roles of Family Cohesion and Parental Dispositional Mindfulness in Picky Eating and Problematic Mealtime Behaviors

**DOI:** 10.3390/bs16101875

**Published:** 2026-10-11

**Authors:** Yingjiao Chen, Ruwen Liang, Limin Zhang

**Affiliations:** 1 School of Education, Guangzhou University, West Waihuan Road 230, Guangzhou 510006, China; 205417@gzhu.edu.cn (Y.C.); zhanglimin333@126.com (L.Z.); 2 Normal College, Jimei University, Yinjiang Road 183, Xiamen 361021, China

**Keywords:** parental phubbing, family cohesion, parental dispositional mindfulness, picky eating, problematic mealtime behaviors

## Abstract

Grounded in family systems theory, this study examined associations between parental phubbing and two forms of preschoolers’ eating problems (picky eating and problematic mealtime behaviors) and the roles of family cohesion and parental dispositional mindfulness in these associations. Using a cross–sectional design, data were collected from 550 parents of preschoolers in Guangzhou, China. In a joint multivariate moderated mediation model, parental phubbing was positively associated with both outcomes at the bivariate level. At mean dispositional mindfulness, its adjusted direct association was significantly stronger for problematic mealtime behaviors than for picky eating. Supplementary simple mediation analyses indicated indirect associations through family cohesion for both outcomes. In the primary moderated model, however, the conditional indirect association through family cohesion was not statistically different from zero for picky eating at low, mean, or high mindfulness, whereas it was evident for problematic mealtime behaviors at mean and high mindfulness. Formal between-outcome differences in the cohesion-related indirect associations and indices of moderated mediation were not significant. Contrary to the hypothesized buffering pattern, higher dispositional mindfulness was associated with a stronger negative phubbing–cohesion association. Overall, the findings highlight modest and differentiated associations between parental phubbing and preschoolers’ eating difficulties and underscore the importance of considering parental technology use within the broader family-relational context.

## 1. Introduction

Early childhood is a critical period for the development of eating patterns, because during these years children’s learning about food and eating helps shape later food choices, diet quality, and weight-related outcomes, and early eating patterns may extend beyond the preschool years (Savage et al., 2007). Eating problems during this stage therefore deserve particular attention, as feeding problems have been estimated to occur in more than 30% of typically developing young children in China (Tang et al., 2023; Xue et al., 2015). Among the various forms of early eating difficulties, the present study focuses on picky eating and problematic mealtime behaviors because they capture two related yet distinguishable aspects of preschoolers’ eating problems. Picky eating primarily concerns food selectivity, refusal, and reluctance to accept a sufficient variety of foods, and has been associated with poorer dietary variety as well as possible concerns about growth and development (Taylor et al., 2015). Problematic mealtime behaviors, by contrast, refer here to difficulties in everyday eating routines, including prolonged meals, holding food in the mouth without swallowing, reliance on television, toys, or stories while eating, and premeal snacking that interferes with regular meals. This distinction is consistent with the Preschooler’s Eating Behaviour Scale used in the present study (Yang et al., 2012) and with pediatric feeding research that treats limited food variety and mealtime behavior problems as separable dimensions of feeding difficulty (Poppert et al., 2015). Focusing on these two outcomes is especially meaningful in preschool years because young children’s eating is closely embedded in family meals and routines, which have been linked to healthier intake, more food enjoyment, and less fussiness (Snuggs & Harvey, 2023; Verhage et al., 2018).

Prior research has linked preschoolers’ eating problems to multiple family–related factors, including parental feeding practices, the organization of family mealtimes, and the quality of parent–child interaction during meals (Bergmeier et al., 2015; Costa & Oliveira, 2023; Snuggs & Harvey, 2023). Parents shape children’s eating not only through specific feeding practices, but also through the broader mealtime environments and interaction patterns they create in everyday family life (Costa & Oliveira, 2023; Verhage et al., 2018). Prospective work further suggests that characteristics of the home feeding environment, including mealtime climate and family routines, are relevant to the development of picky eating in preschool–aged children (Cole et al., 2018). Less attention has been given to the role of digital device use within these family interactions. Research on parental technoference indicates that parents’ technology use can interrupt parent–child exchanges and is associated with parent–child relationship quality as well as children’s health and developmental outcomes (Komanchuk et al., 2023). One increasingly common manifestation of such disruption is parental phubbing, defined as parents’ smartphone use in the presence of their children in ways that convey inattention or social disengagement (Pancani et al., 2021). Although parental phubbing has been linked to adverse child adjustment, its association with Chinese preschoolers’ eating problems remains comparatively underexplored, particularly from a family-relational perspective (Zhang et al., 2023).

Parental phubbing can be situated within family systems theory, which conceptualizes the family as a set of interdependent subsystems and views child development as embedded in ongoing transactions across family relationships (Cox & Paley, 1997). From this perspective, parental phubbing is relevant not only as an individual smartphone–use behavior but also as a feature of everyday parent–child interaction. Phubbing signals inattention and has been associated with children’s feelings of social disconnection from parents (Komanchuk et al., 2023; Pancani et al., 2021). This lens is especially relevant to preschoolers’ eating because feeding in early childhood is embedded in reciprocal parent–child exchanges. Responsive feeding emphasizes prompt, emotionally supportive, and contingent caregiver responses to children’s cues, and research on family mealtimes similarly links responsive feeding and regular shared meals with healthier eating-related outcomes in children (Black & Aboud, 2011; Killion et al., 2024; Wen et al., 2025). When smartphone use competes with attention during routine family interactions, including mealtimes, opportunities to notice children’s signals, provide guidance, and sustain engagement may be reduced. Parental phubbing is therefore a plausible relational correlate of both picky eating and problematic mealtime behaviors. Accordingly, we proposed the following hypotheses:

**H1a.**  *At the bivariate level, parental phubbing would be positively associated with preschoolers’ picky eating.*

**H1b.**  *At the bivariate level, parental phubbing would be positively associated with preschoolers’ problematic mealtime behaviors.*

Within this family-relational perspective, family cohesion is a relevant family–level construct. In the present study, family cohesion is treated as a continuous indicator of perceived emotional bonding and connectedness among family members, consistent with the Chinese FACES II cohesion subscale used in this study (Fei et al., 1991). Recent evidence has linked higher parental phubbing with lower family cohesion, suggesting that parental smartphone–related inattention may be associated with the broader relational climate of family life (Yu et al., 2025). Family cohesion is also relevant to preschoolers’ eating because mealtimes depend on coordination, responsiveness, and predictable family interaction. Research has shown that family functioning, including cohesion and conflict, is associated with food–related behaviors in preschool families, and that a more positive mealtime emotional climate is associated with better nutrition and fewer behavioral difficulties in children (Martin-Biggers et al., 2017; Rosemond et al., 2019). Related evidence further suggests that supportive family-level processes, such as dietary coparenting and constructive parenting strategies, are associated with fewer picky eating behaviors in young children (J.-L. Chen et al., 2024). Taken together, this literature provides a basis for examining whether parental phubbing is indirectly associated with preschoolers’ eating problems through family cohesion. Accordingly, we proposed the following hypotheses:

**H2a.**  *Parental phubbing would show an indirect association with preschoolers’ picky eating through family cohesion.*

**H2b.**  *Parental phubbing would show an indirect association with preschoolers’ problematic mealtime behaviors through family cohesion.*

Parental dispositional mindfulness may further shape the strength of these associations. Dispositional mindfulness refers to a general tendency to attend to and remain aware of present–moment experience in everyday life (Brown & Ryan, 2003). This construct is distinct from mindful parenting, which refers specifically to mindful awareness, emotional regulation, and responsiveness within parent–child interactions (Duncan et al., 2009). Family systems perspectives further suggest that associations between relational disruptions and family outcomes need not be uniform across families, because parents may differ in the attentional and self–regulatory resources they bring to everyday family interactions (Cox & Paley, 1997). Evidence from Chinese families nevertheless suggests that greater parental dispositional mindfulness is associated with more adaptive parenting processes and child adjustment (Han et al., 2021; Yan et al., 2021), and recent work has examined mindful attention awareness in relation to parental phubbing and preschoolers’ behavioral problems (Puligheddu et al., 2025). These findings provide a basis for considering dispositional mindfulness as a general attentional and self–regulatory resource that may shape how strongly phubbing is associated with family relationships and child outcomes. At a comparable level of phubbing, parents higher in dispositional mindfulness may be better able to maintain awareness of relational cues and regulate attention during everyday family interactions. We therefore expected higher dispositional mindfulness to attenuate the negative association between phubbing and family cohesion, as well as the direct and cohesion-related indirect associations with preschoolers’ eating problems.

**H3.**  *Parental dispositional mindfulness would moderate the association between parental phubbing and family cohesion, such that the negative association would be weaker at higher levels of parental dispositional mindfulness.*

**H4a.**  *Parental dispositional mindfulness would moderate the direct association between parental phubbing and preschoolers’ picky eating, such that the association would be weaker at higher levels of parental dispositional mindfulness.*

**H4b.**  *Parental dispositional mindfulness would moderate the direct association between parental phubbing and preschoolers’ problematic mealtime behaviors, such that the association would be weaker at higher levels of parental dispositional mindfulness.*

**H5a.**  *The indirect association between parental phubbing and preschoolers’ picky eating through family cohesion would be weaker at higher levels of parental dispositional mindfulness.*

**H5b.**  *The indirect association between parental phubbing and preschoolers’ problematic mealtime behaviors through family cohesion would be weaker at higher levels of parental dispositional mindfulness.*

### The Present Study

**Figure 1:**
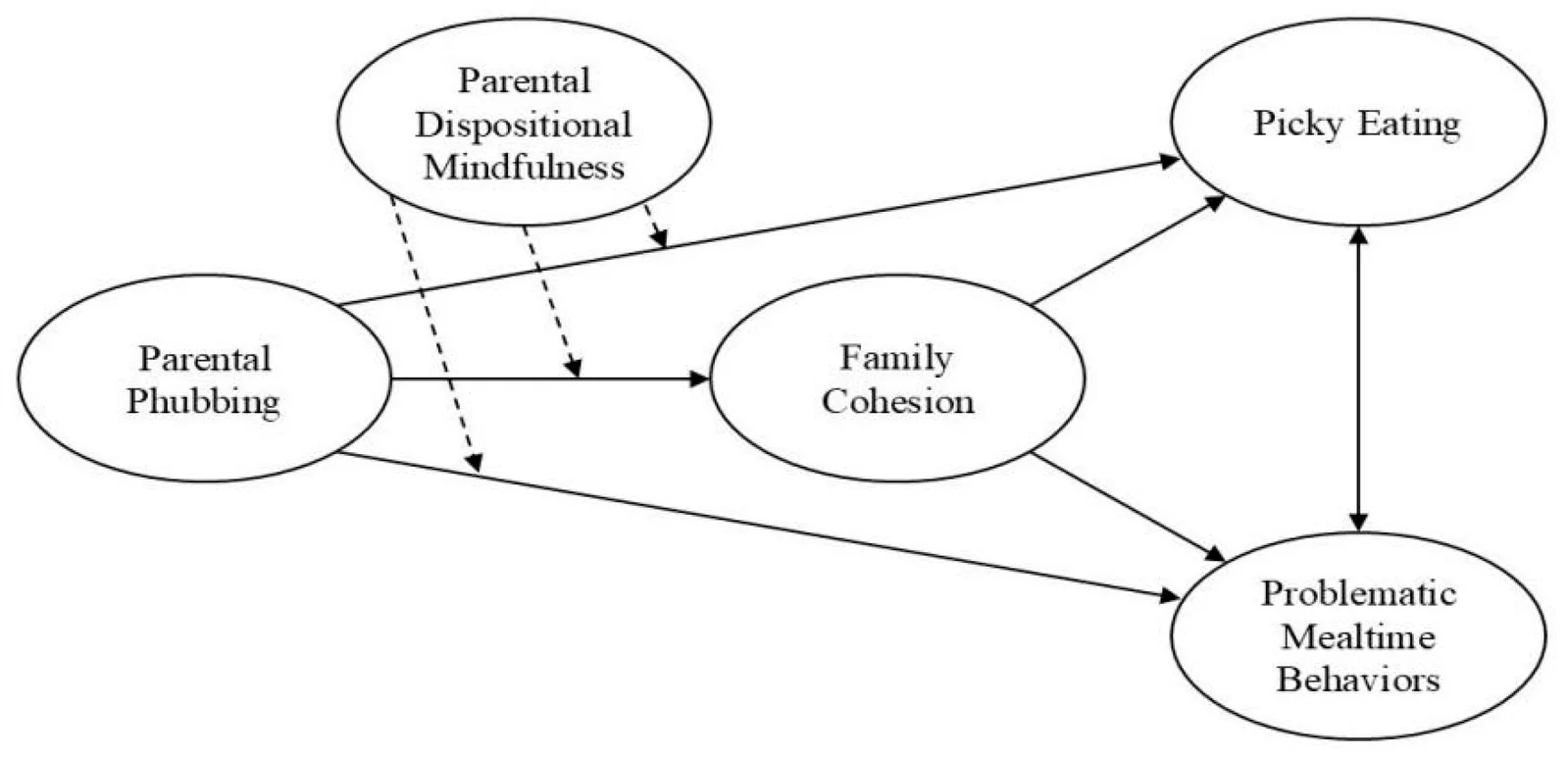
Hypothetical model. Note. Solid single-headed arrows represent hypothesized regression paths; dashed arrows indicate hypothesized moderation of the corresponding paths by parental dispositional mindfulness. The solid double-headed arrow represents the residual covariance between the two eating outcomes. Arrows specify the statistical model and do not imply causality.

Against this background, the present study examined cross–sectional associations among parental phubbing, family cohesion, parental dispositional mindfulness, and two related but distinguishable forms of preschoolers’ eating problems: picky eating and problematic mealtime behaviors. Drawing on family systems theory, we evaluated whether parental phubbing was indirectly associated with each eating outcome through family cohesion and whether parental dispositional mindfulness moderated the phubbing–cohesion association, the direct phubbing–outcome associations, and the corresponding conditional indirect associations (Figure 1). Because the two eating outcomes were conceptually related and empirically correlated (Yang et al., 2012), they were modeled jointly so that corresponding paths could be formally compared across outcomes. In doing so, the present study extends research on parental phubbing beyond predominantly social–emotional outcomes and examines how digital–age family interaction may be associated with preschoolers’ eating difficulties within a broader family–relational context.

## 2. Materials and Methods

### 2.1. Participants and Procedure

Ethics approval for this study was granted by the Human Research Ethics Committee (HREC) of Guangzhou University prior to data collection (Date: 20 May 2023; No. GZHU202342). Data were collected in September 2024. Before data collection, the target sample size was informed by simulation–based guidance for mediation analysis. Fritz and MacKinnon (2007) reported that, under relatively small component-path effects, approximately 462 participants may be required to achieve 80% power when testing an indirect effect using a bias-corrected bootstrap procedure. Because the present study involved a moderated mediation model, multiple covariates, and two correlated outcomes, we used this value as a conservative literature–based benchmark rather than as an exact power calculation for the present model. We therefore aimed to recruit more than 462 participants.

Participants were recruited using convenience sampling in five preschools in Guangzhou, Guangdong Province, China. Before data collection, we contacted preschool principals to obtain support for the survey. After receiving approval, kindergarten representatives distributed the digital survey link to parents through WeChat class groups. Parents were informed of the purpose of the study and provided informed consent before participating voluntarily. To ensure independent responses, only one parent as the primary caregiver (either the father or the mother) of each child was invited to complete the questionnaires.

A total of 601 questionnaires were submitted. We applied several prespecified data–quality screening criteria. Prior to the main survey, the questionnaire was pilot-tested with 30 parents from the target population using the same online administration format, consistent with commonly cited recommendations for preliminary questionnaire pilot testing (Johanson & Brooks, 2010). The median completion time in the pilot was 810 s. Guided by the relative completion-speed approach proposed by Leiner (2019), we defined unusually rapid completion as less than one third of the pilot median, yielding a screening threshold of 270 s. 32 records were excluded because the completion time was less than 270 s. Second, we excluded questionnaires showing patterned responding (i.e., all selected options in one scale are identical). This criterion was used to identify potentially inattentive responding. 19 additional records were excluded for patterned responding, defined as selecting the same response option for every item within a scale. Third, the online survey system required all items to be completed before submission. As a result, submitted questionnaires contained no item–level missing data. In total, 51 unique records were excluded, leaving 550 parents in the final analytic sample. Detailed demographic characteristics of the participants are presented in Table 1.

### 2.2. Measures

#### 2.2.1. Parental Phubbing Scale

Parental phubbing was measured by the Chinese version of the Parental Phubbing Scale (PPS; Ding et al., 2018), originally developed by Roberts and David (2016). This scale is unidimensional and consists of nine items (e.g., “When I eat with my child, I use my mobile phone.”) All items are rated by parents on a 5-point Likert scale ranging from 1 (“never”) to 5 (“always”), with higher scores indicating more frequent phubbing behavior in daily life. In this study, the scale demonstrated acceptable reliability (Cronbach’s *α* = 0.782).

#### 2.2.2. Mindful Attention Awareness Scale

Parental dispositional mindfulness was assessed using the Chinese version of the Mindful Attention Awareness Scale (MAAS; S. Chen et al., 2012), originally developed by Brown and Ryan (2003). Parents completed this 15-item unidimensional scale (e.g., “I find myself doing things without paying attention.”) using a 6-point Likert scale ranging from 1 (“always”) to 6 (“never”), with higher mean scores indicating greater dispositional mindfulness. The scale showed strong internal consistency (Cronbach’s *α* = 0.920).

#### 2.2.3. Family Cohesion Questionnaire

The study used the cohesion subscale of the Chinese adaptation of FACES II (Fei et al., 1991) as a continuous measure of perceived emotional bonding. Parents completed this 16-item subscale (e.g., “The relationship among family members is very close.”) using a 5-point Likert scale ranging from 1 (“never”) to 5 (“always”). Higher scores indicated stronger family cohesion. The subscale showed strong internal consistency (Cronbach’s *α* = 0.900).

#### 2.2.4. Preschoolers’ Eating Behavior Questionnaire

Two subscales of the Chinese Preschoolers’ Eating Behavior Questionnaire (Yang et al., 2012) were employed to assess child picky eating and problematic mealtime behaviors. The Picky Eating subscale consists of seven items (e.g., “My child refuses many foods because of their smell, taste, appearance, or texture.”) while the Problematic Mealtime Behaviors subscale includes five items (e.g., “My child takes a long time to eat, exceeding 30 min.”) Parents rated their child’s behaviors on a 5-point Likert scale ranging from 1 (“never”) to 5 (“always”), with higher scores indicating more frequent picky eating and problematic mealtime behaviors. The two subscales showed acceptable internal consistency (Cronbach’s *α* = 0.737 and 0.756, respectively).

### 2.3. Statistical Analysis

All analyses were conducted in R. Preliminary analyses included descriptive statistics, Pearson correlations, and measurement checks. Because a central contribution of the study concerns the distinction between picky eating and problematic mealtime behaviors, an item–level confirmatory factor analysis (CFA) compared the hypothesized two-factor model with a one–factor alternative. A second targeted CFA compared parental phubbing and parental dispositional mindfulness as two correlated factors with a one-factor alternative. The five-point and six-point item responses were treated as approximately continuous and the models were estimated using maximum likelihood. Model fit was evaluated jointly using chi–square, CFI, TLI, RMSEA with 90% confidence intervals, and SRMR rather than relying on a single rigid pass/fail cutoff (Hu & Bentler, 1999; Marsh et al., 2004). Composite reliability (CR) values of 0.70 or higher and average variance extracted (AVE) values of 0.50 or higher were used as conventional reference values for reliability and convergent validity, respectively (Fornell & Larcker, 1981). For discriminant validity, HTMT values below 0.85 were treated as conservative evidence that paired constructs were empirically distinguishable (Henseler et al., 2015). Latent factor correlations were also inspected. Full measurement results are reported in Appendix A Table A1, Table A2 and Table A3. Harman’s single-factor test was then retained as a descriptive diagnostic of potential shared-method structure.

The primary hypotheses were evaluated in a joint multivariate moderated mediation framework. Parental phubbing was specified as the predictor (X), family cohesion as the intervening variable (M), and parental dispositional mindfulness as the moderator (W). Picky eating and problematic mealtime behaviors were modeled simultaneously as correlated outcomes, with their residual covariance freely estimated. The same six demographic covariates used in the original analysis plan were retained in the mediator and outcome equations: primary caregiver/respondent (father vs. mother), child sex, child age, only–child status, household registration, and household annual income. These covariates were selected a priori because prior research indicates that child and family demographic characteristics can be associated with children’s eating behavior and related parenting or family contexts (Cai et al., 2018; Keller et al., 2019; Scaglioni et al., 2018). Parental phubbing and parental dispositional mindfulness were mean-centered before constructing the interaction term. Regression coefficients are reported as unstandardized coefficients (*b*) with HC3 heteroskedasticity-consistent standard errors and 95% confidence intervals; HC3 was used to reduce sensitivity to heteroskedasticity in regression inference (Long & Ervin, 2000).

Conditional indirect associations were evaluated with 5000 case–resampling bootstrap samples at low (−1 *SD*), mean, and high (+1 *SD*) levels of parental dispositional mindfulness. A 95% bootstrap confidence interval excluding zero was interpreted as evidence that the corresponding indirect association was statistically different from zero. Moderated mediation was evaluated using the index of moderated mediation, defined as the product of the first-stage interaction coefficient and the outcome–specific family–cohesion coefficient; inference was based on the bootstrap confidence interval for the index rather than on comparing the significance of conditional indirect effects across moderator values (Hayes, 2015). Formal between–outcome tests were used to compare the corresponding direct phubbing paths, family–cohesion paths, direct interaction paths, conditional indirect associations, and indices of moderated mediation while accounting for the correlation between the two outcomes.

To facilitate comparison with the original simple mediation analyses, a supplementary joint simple mediation model was also estimated in which the two eating outcomes were modeled simultaneously without the moderation terms. Indirect associations were again evaluated using 5000 bootstrap samples. Simple–slope and Johnson–Neyman analyses were treated as follow–up probes of the significant first-stage interaction rather than as separate confirmatory tests. To address multiplicity, Holm corrections were applied to the prespecified hypothesis-level tests and, separately, to the formal between–outcome comparisons (Holm, 1979). 

### 2.4. Use of Generative AI in Manuscript Preparation

The authors used ChatGPT (OpenAI; model version: GPT-5.4 Thinking) for limited language refinement during manuscript preparation. The tool was used only to improve the clarity, grammar, readability, and fluency of selected sentences and paragraphs. It was not used to generate research ideas, design the study, analyze data, interpret results, or draw conclusions. The authors reviewed and edited all AI-assisted language changes and take full responsibility for the final content of the manuscript.

## 3. Results

### 3.1. Measurement and Common-Method Considerations

The targeted CFAs provided evidence that the focal constructs could be empirically distinguished, although absolute fit was not uniformly strong. For the child eating outcomes, the hypothesized two-factor model fit substantially better, *χ*^2^(53) = 244.98, *p* < 0.001, CFI = 0.873, TLI = 0.842, RMSEA = 0.081, 90% CI [0.071, 0.092], SRMR = 0.073, than the one-factor alternative, *χ*^2^(54) = 503.07, *p* < 0.001, CFI = 0.704, TLI = 0.638, RMSEA = 0.123, 90% CI [0.113, 0.133], SRMR = 0.097. The two latent eating factors were moderately correlated (*r* = 0.53), and their HTMT ratio was 0.60. Similarly, the two–factor model distinguishing parental phubbing from parental dispositional mindfulness fit substantially better, *χ*^2^(251) = 893.67, *p* < 0.001, CFI = 0.888, TLI = 0.876, RMSEA = 0.068, 90% CI [0.063, 0.073], SRMR = 0.051, than the corresponding one-factor model, *χ*^2^(252) = 1726.72, *p* < 0.001, CFI = 0.742, TLI = 0.717, RMSEA = 0.103, 90% CI [0.099, 0.108], SRMR = 0.095. The latent correlation between parental phubbing and parental dispositional mindfulness was −0.53, and HTMT was 0.56. Full loadings and measurement indices are reported in Appendix A Table A1, Table A2 and Table A3.

Because all focal variables were reported by the same parent at the same assessment occasion, shared-method variance remained a potential concern. Harman’s single–factor test yielded 11 factors with eigenvalues greater than 1, and the first factor accounted for 22.73% of the total variance. Although this diagnostic did not indicate that one factor dominated the item covariance, it cannot determine the magnitude of common-method bias or establish that such bias is negligible. Some shared–method influence may remain, and the findings should therefore be interpreted cautiously as associations that may partly reflect common reporting tendencies.

### 3.2. Descriptive Statistics and Correlations

Descriptive statistics and zero–order correlations among the focal variables are shown in Table 2. Parental phubbing was positively associated with picky eating, *r* = 0.21, *p* < 0.001, and problematic mealtime behaviors, *r* = 0.28, *p* < 0.001, supporting H1a and H1b at the bivariate level. Parental phubbing was negatively associated with family cohesion and parental dispositional mindfulness, whereas family cohesion and dispositional mindfulness were each negatively associated with both eating outcomes.

### 3.3. Primary Joint Moderated Mediation Model

The primary joint model (Table 3) simultaneously estimated picky eating and problematic mealtime behaviors while allowing their residuals to covary; the residual correlation between the two outcome equations was 0.38. In the family-cohesion equation, parental phubbing was negatively associated with family cohesion, *b* = −0.130, HC3 *SE* = 0.046, *p* = 0.004, whereas parental dispositional mindfulness was positively associated with family cohesion, *b* = 0.292, HC3 *SE* = 0.038, *p* < 0.001. The phubbing × dispositional mindfulness interaction was significant, *b* = −0.153, HC3 *SE* = 0.036, *p* < 0.001. Because the negative phubbing-cohesion association became stronger, rather than weaker, as mindfulness increased, the observed interaction was opposite to the buffering pattern predicted in H3; thus, H3 was not supported.

For picky eating, neither the direct association of parental phubbing, *b* = 0.068, HC3 *SE* = 0.047, *p* = 0.148, nor the family-cohesion path, *b* = −0.073, HC3 *SE* = 0.048, *p* = 0.126, was statistically significant. Parental dispositional mindfulness was negatively associated with picky eating, *b* = −0.187, HC3 *SE* = 0.038, *p* < 0.001, whereas the direct phubbing × mindfulness interaction was not significant, *b* = −0.018, HC3 *SE* = 0.034, *p* = 0.603. For problematic mealtime behaviors, parental phubbing remained positively associated with the outcome, *b* = 0.245, HC3 *SE* = 0.064, *p* < 0.001, and family cohesion was negatively associated with the outcome, *b* = −0.165, HC3 *SE* = 0.055, *p* = 0.003. Parental dispositional mindfulness was also negatively associated with problematic mealtime behaviors, *b* = −0.135, HC3 *SE* = 0.045, *p* = 0.003, whereas the direct phubbing × mindfulness interaction was not significant, *b* = 0.007, HC3 *SE* = 0.052, *p* = 0.894. Thus, H4a and H4b were not supported.

Formal between-outcome tests showed that the direct phubbing coefficient was significantly larger for problematic mealtime behaviors than for picky eating at the mean level of dispositional mindfulness, Δ*b* = 0.177, *SE* = 0.055, *z* = 3.24, *p* = 0.001, 95% CI [0.070, 0.284]. In contrast, the difference between the two family-cohesion coefficients was not statistically significant, Δ*b* = −0.092, *SE* = 0.053, *z* = −1.74, *p* = 0.082, nor was the difference between the two direct phubbing × mindfulness interaction coefficients, Δ*b* = 0.025, *SE* = 0.054, *z* = 0.46, *p* = 0.646. Detailed between-outcome comparisons are reported in Appendix A Table A4.

### 3.4. Conditional Indirect Associations and Moderated Mediation

For picky eating, the conditional indirect association through family cohesion was not statistically different from zero at low dispositional mindfulness, *ab* = 0.001, 95% bootstrap CI [−0.009, 0.010], at the mean, *ab* = 0.009, 95% CI [−0.002, 0.025], or at high dispositional mindfulness, *ab* = 0.018, 95% CI [−0.005, 0.045]. The index of moderated mediation was 0.011, 95% bootstrap CI [−0.003, 0.028], providing no evidence of moderated mediation for picky eating; H5a was therefore not supported.

For problematic mealtime behaviors, the conditional indirect association was not statistically different from zero at low dispositional mindfulness, *ab* = 0.001, 95% bootstrap CI [−0.017, 0.018], but was positive at the mean, *ab* = 0.021, 95% CI [0.004, 0.044], and at high dispositional mindfulness, *ab* = 0.041, 95% CI [0.013, 0.075]. The index of moderated mediation was 0.025, 95% bootstrap CI [0.008, 0.048], indicating that the indirect association varied across levels of dispositional mindfulness. However, the conditional indirect association became stronger rather than weaker as mindfulness increased; accordingly, H5b was not supported in the hypothesized direction.

Importantly, the difference between the two outcome-specific indices of moderated mediation was not statistically different from zero, Δindex = 0.014, 95% bootstrap CI [−0.002, 0.032]. Likewise, the between-outcome differences in the conditional indirect associations were not statistically significant at low, mean, or high levels of the moderator. Thus, the presence of a statistically detectable moderated indirect association for problematic mealtime behaviors, but not for picky eating, should not be interpreted as evidence that the two moderated indirect effects differed significantly from one another. Detailed between-outcome comparisons are shown in Appendix A Table A4, and outcome-specific indirect associations and indices are shown in Table A5.

### 3.5. Supplementary Joint Simple Mediation Analysis

A supplementary joint simple mediation analysis was conducted to estimate the average indirect associations without the moderation terms. Parental phubbing was negatively associated with family cohesion, *b* = −0.253, HC3 *SE* = 0.041, *p* < 0.001. The indirect association through family cohesion was statistically different from zero for picky eating, *ab* = 0.036, 95% bootstrap CI [0.013, 0.063], and for problematic mealtime behaviors, *ab* = 0.055, 95% bootstrap CI [0.027, 0.086]. However, the difference between the two indirect associations was not statistically significant, Δ*ab* = 0.019, 95% bootstrap CI [−0.006, 0.045]. The family–cohesion coefficients in the two outcome equations also did not differ significantly from one another, Δ*b* = −0.075, *p* = 0.135. These supplementary findings were consistent with H2a and H2b at the level of average indirect associations, although the primary moderated model indicated that these associations were conditional on dispositional mindfulness. Full results are reported in Appendix A Table A6.

### 3.6. Follow-Up Probes and Multiplicity Checks

Follow-up probing of the significant phubbing × dispositional mindfulness interaction in the family–cohesion equation showed that the conditional phubbing slope was not significant at low mindfulness (−1 *SD*), *b* = −0.008, HC3 *SE* = 0.052, *p* = 0.870, but was negative at the mean, *b* = −0.130, HC3 *SE* = 0.046, *p* = 0.004, and at high mindfulness (+1 *SD*), *b* = −0.251, HC3 *SE* = 0.056, *p* < 0.001. To identify the range of dispositional mindfulness over which the conditional phubbing slope was statistically distinguishable from zero and to describe sample coverage across those regions, we conducted a Johnson–Neyman analysis. The analysis identified raw mindfulness values of approximately 2.71 and 4.28 as transition points. The conditional association was statistically different from zero below 2.71 and above 4.28, but not between these values. Only 6 participants (1.1%) fell below 2.71, 191 (34.7%) fell between 2.71 and 4.28, and 353 (64.2%) were above 4.28. Because the region below 2.71 contained very few observations, no substantive interpretation was assigned to the association in that region. Full probe results are reported in Appendix A Table A7.

Holm corrections were applied to the prespecified hypothesis-level tests and, separately, to the formal between-outcome comparisons. The correction did not change the substantive pattern of conclusions. In particular, the between–outcome difference in the direct phubbing path remained statistically significant after adjustment, whereas the differences in the cohesion-related paths, conditional indirect associations, and moderated mediation indices did not. Full adjusted results are reported in Appendix A Table A8.

## 4. Discussion

The present study examined cross–sectional associations among parental phubbing, family cohesion, parental dispositional mindfulness, and two related but distinguishable forms of preschoolers’ eating problems. Three findings stand out. First, parental phubbing was positively associated with both picky eating and problematic mealtime behaviors at the bivariate level. In the primary joint model, however, the direct association with problematic mealtime behaviors was significantly stronger than the corresponding association with picky eating at the mean level of dispositional mindfulness. Second, family cohesion was involved in indirect associations with both outcomes in the supplementary simple mediation model, but the primary moderated model did not establish a stronger cohesion-related pathway for one outcome than the other. Third, parental dispositional mindfulness did not show the hypothesized buffering pattern. The phubbing × dispositional mindfulness interaction for family cohesion was significant in the opposite direction to that predicted, and the index of moderated mediation was significant for problematic mealtime behaviors but likewise opposite to the hypothesized direction. These findings refine, rather than overturn, the broader expectation that digital distraction, family relational functioning, and children’s eating difficulties are interrelated.

The distinction between picky eating and problematic mealtime behaviors remains an important feature of the findings. The targeted CFA supported treating the two sets of eating items as related but distinguishable constructs, and their zero–order correlation indicated substantial shared variance without suggesting interchangeability. At the bivariate level, parental phubbing was associated with both outcomes, consistent with the idea that parents’ technology-related distraction may covary with the broader family context in which young children eat (Bergmeier et al., 2015; Snuggs & Harvey, 2023; Verhage et al., 2018). In the joint model, the direct phubbing coefficient for problematic mealtime behaviors was larger than the corresponding coefficient for picky eating, and this difference was formally significant. One plausible interpretation is that behaviors such as prolonged meals, distraction during eating, and difficulty sustaining mealtime routines are especially embedded in ongoing parent–child coordination, whereas picky eating also reflects food selectivity and other child-level characteristics that may be less directly tied to moment-to-moment family interaction (Poppert et al., 2015; Taylor et al., 2015). This interpretation should be understood as a difference in the observed pattern of association, not as evidence that parental phubbing uniquely determines mealtime behavior problems.

The findings for family cohesion were more nuanced than the original formulation suggested. In the supplementary joint simple mediation model, parental phubbing showed significant indirect associations with both picky eating and problematic mealtime behaviors through family cohesion. In the primary moderated model, the conditional indirect association was not statistically different from zero for picky eating at low, mean, or high dispositional mindfulness, whereas it was detectable for problematic mealtime behaviors at mean and high mindfulness. However, formal between–outcome tests did not show significant differences in the family–cohesion b-paths, the conditional indirect associations, or the indices of moderated mediation. Accordingly, the present data do not support the stronger claim that family cohesion is a more powerful or more proximal relational process for problematic mealtime behaviors than for picky eating. A more defensible interpretation is that family cohesion is relevant to the broader family context of both forms of eating difficulty, while the strength and detectability of its indirect association vary across model specifications and levels of dispositional mindfulness. This interpretation is consistent with research linking family cohesion, mealtime climate, and family interaction to children’s food–related behavior (Martin-Biggers et al., 2017; Rosemond et al., 2019).

The MAAS used in this study assesses a general tendency toward attentive awareness in everyday life rather than mindfulness specifically enacted in parenting interactions (Brown & Ryan, 2003). Mindful parenting is a related but more context-specific construct involving attention, emotional awareness, and self-regulation in interactions with one’s child (Duncan et al., 2009). The study therefore tested dispositional mindfulness as a potential boundary condition, not mindful parenting itself. Contrary to H3, higher dispositional mindfulness did not weaken the negative association between parental phubbing and family cohesion; the observed interaction was in the opposite direction. H4a and H4b were not supported because dispositional mindfulness did not moderate the direct phubbing associations with either eating outcome. H5a was also not supported, whereas the moderated mediation index for problematic mealtime behaviors was statistically different from zero but in the direction opposite to H5b.

This unexpected pattern should not be interpreted as evidence that dispositional mindfulness is harmful or that it amplifies the effects of parental phubbing. The result is better viewed as an exploratory finding showing that the association between phubbing and perceived family cohesion differed across levels of general dispositional mindfulness in this sample. One possibility is that general attentional awareness changes how parents perceive or report relational disruptions in everyday family life. At the same time, all focal constructs were reported by the same parent at one occasion, and both the phubbing and mindfulness measures contain attentional content. Although the targeted CFA and HTMT results supported their empirical distinguishability, these design and measurement features remain relevant when interpreting an interaction that was opposite to prediction. Replication with longitudinal designs, parenting–specific mindfulness measures, and multi–informant or observational data would be especially useful before assigning a stronger theoretical meaning to this pattern.

The magnitude of the findings should also be kept in perspective. The primary outcome models explained approximately 12.3% of the variance in picky eating and 13.8% of the variance in problematic mealtime behaviors, and the conditional indirect associations were small in absolute size. With a sample of 550 participants, modest effects can be estimated with reasonable precision, but statistical detectability should not be equated with large practical impact. The contribution of the present study therefore lies primarily in clarifying the pattern of associations across two eating constructs, formally comparing corresponding paths, and identifying questions for future family–process research rather than in demonstrating a large predictive effect of parental phubbing or dispositional mindfulness.

### 4.1. Limitations

Several limitations should be acknowledged. First, the cross-sectional design does not establish temporal ordering or causality. The indirect associations reported here are therefore statistical decompositions of contemporaneous associations rather than evidence that parental phubbing causes changes in family cohesion that subsequently produce children’s eating problems. Reciprocal processes are plausible; for example, difficult mealtimes may increase parental stress, alter perceptions of family functioning, or change parents’ patterns of phone use during daily routines.

Second, all focal variables were reported by the same parent at the same assessment occasion. Shared–method variance and reporter-specific response tendencies therefore cannot be ruled out. Harman’s single-factor test was retained as a descriptive diagnostic, but it does not resolve this concern. Future studies would benefit from combining parent reports with co-parent or teacher reports, direct mealtime observation, passive or ecological measures of smartphone use, and repeated assessments of children’s eating behavior.

Third, the sample was obtained by convenience sampling from five preschools in Guangzhou and was predominantly composed of mothers and urban families. The findings therefore should not be assumed to generalize to fathers, rural families, other regions of China, or families with different socioeconomic backgrounds. More diverse samples would also allow direct examination of whether these associations vary across caregiver and family contexts.

Finally, the measurement results supported the intended distinctions between the two eating outcomes and between parental phubbing and dispositional mindfulness, but model fit and convergent–validity indices were not uniformly strong. In addition, the MAAS measures general dispositional mindfulness rather than parenting-specific mindfulness. Future studies could compare dispositional mindfulness and mindful parenting within the same model to determine whether they show different relations with parental technology use and family processes.

### 4.2. Implications

Theoretically, the findings support distinguishing between different forms of preschoolers’ eating difficulty rather than treating eating problems as a single undifferentiated category. The formal comparison of the two outcomes suggests that parental phubbing may be more closely associated with problematic mealtime behaviors at the level of the direct adjusted association, while the evidence did not establish a corresponding between–outcome difference in cohesion-related indirect processes. This pattern is compatible with a family-systems perspective in which digital device use is one element of a broader interactional context rather than a stand-alone determinant of child behavior.

The findings also suggest that family cohesion is worth retaining in models of children’s eating because it captures a broader relational context that covaries with both parental phubbing and eating difficulties. At the same time, the present results argue against assigning family cohesion a uniquely stronger mediating role for one eating outcome without a direct statistical comparison. Future work could examine more proximal mealtime processes, such as caregiver responsiveness, consistency of guidance, conflict, and routine organization, alongside broader family cohesion to clarify which relational processes are most closely linked to specific eating behaviors.

For applied work, parental device use during family meals may be a useful topic for assessment and future intervention research, particularly when the presenting concern involves mealtime routines or behavior. However, the present cross-sectional findings do not demonstrate that reducing phubbing would by itself improve children’s eating. Likewise, the unexpected dispositional-mindfulness findings do not support using mindfulness as a simple universal buffer. Intervention studies that directly manipulate technology routines or evaluate parenting-specific mindfulness would be needed to determine whether changing these factors produces meaningful changes in family interaction or child eating behavior.

## 5. Conclusions

Parental phubbing was positively associated with both picky eating and problematic mealtime behaviors, but its adjusted direct association was significantly stronger for problematic mealtime behaviors in the primary joint model. In the primary moderated model, the conditional indirect association through family cohesion was not statistically significant for picky eating at low, mean, or high dispositional mindfulness, whereas it was statistically significant for problematic mealtime behaviors at mean and high mindfulness. However, formal comparisons did not establish between-outcome differences in these indirect associations or in the indices of moderated mediation. The supplementary simple model indicated average indirect associations through family cohesion for both outcomes. Parental dispositional mindfulness did not show the hypothesized buffering pattern, and the opposite-direction phubbing–cohesion interaction should be interpreted cautiously pending replication. Given the modest effect sizes and cross-sectional design, the findings should be interpreted as patterns of association rather than evidence of causal pathways.

## Figures and Tables

**Table 1:** Demographic characteristics of the participants.

Characteristic	Category	*n*	%	*M* (*SD*)
Primary caregiver	Mother	430	78.18	
	Father	120	21.82	
Child sex	Boy	288	52.36	
	Girl	262	47.64	
Child age (years)				4.80 (1.01)
Only–child status	Only child	134	24.36	
	Non–only child	416	75.64	
Household registration	Rural	82	14.91	
	Urban	468	85.09	
Household annual income	0–100,000 CNY	94	17.09	
	100,001–500,000 CNY	330	60.00	
	Above 500,000 CNY	126	22.91	

**Table 2:** Descriptive statistics and correlations between variables.

Variable	*M*	*SD*	1	2	3	4	5	6	7	8	9	10	11
1. Primary caregiver			1										
2. Child sex			0.06	1									
3. Child age			0.01	−0.03	1								
4. Only–child status			0.05	0.02	0.08	1							
5. Household registration			−0.15 ***	−0.05	−0.05	0.00	1						
6. Household annual income			−0.02	0.12 **	0.02	0.03	−0.23 ***	1					
7. Parental phubbing	2.54	0.60	0.01	−0.10 *	0.02	−0.08	0.08 *	−0.13 **	1				
8. Family cohesion	3.75	0.61	0.02	0.05	−0.01	0.02	−0.09 *	0.11 *	−0.26 ***	1			
9. Parental dispositional mindfulness	4.54	0.79	0.00	0.04	−0.08	0.04	−0.11 *	0.06	−0.46 ***	0.40 ***	1		
10. Picky eating	2.78	0.60	−0.03	0.01	−0.04	−0.04	0.12 **	−0.00	0.21 ***	−0.19 ***	−0.31 ***	1	
11. Problematic mealtime behaviors	2.34	0.77	−0.10 *	−0.08	−0.04	−0.00	0.03	−0.02	0.28 ***	−0.24 ***	−0.27 ***	0.44 ***	1

Note. Primary caregiver: 0 = father, 1 = mother; Child sex: 0 = boy, 1 = girl; Only–child status: 0 = only child, 1 = non-only child; Household registration: 0 = rural, 1 = urban; Household annual income: 0 = 0–100,000 CNY, 1 = 100,001–500,000 CNY, 2 = above 500,000 CNY. * *p* < 0.05. ** *p* < 0.01. *** *p* < 0.001 (two–tailed).

**Table 3:** Joint Multivariate Moderated Mediation Model for Preschoolers’ Eating Problems.

Predictor	*b*	HC3 *SE*	95% CI	*p*
Panel A. Family cohesion equation				
Primary caregiver	0.033	0.061	[−0.086, 0.153]	0.585
Child sex	0.014	0.048	[−0.081, 0.109]	0.767
Child age	0.008	0.023	[−0.036, 0.053]	0.718
Only–child status	−0.029	0.057	[−0.141, 0.083]	0.612
Household registration	0.054	0.072	[−0.087, 0.196]	0.450
Household annual income	0.079	0.042	[−0.004, 0.162]	0.063
Parental phubbing (*X*)	−0.130	0.046	[−0.219, −0.040]	0.004
Dispositional mindfulness (*W*)	0.292	0.038	[0.217, 0.366]	<0.001
*X* × *W*	−0.153	0.036	[−0.223, −0.082]	<0.001
*R* ^2^	0.199			
Panel B. Picky eating equation				
Primary caregiver	−0.028	0.053	[−0.132, 0.075]	0.593
Child sex	0.036	0.049	[−0.059, 0.131]	0.460
Child age	−0.034	0.020	[−0.073, 0.005]	0.089
Only–child status	−0.027	0.058	[−0.141, 0.086]	0.638
Household registration	−0.153	0.075	[−0.299, −0.007]	0.039
Household annual income	0.049	0.043	[−0.035, 0.134]	0.255
Parental phubbing (*X*)	0.068	0.047	[−0.024, 0.161]	0.148
Family cohesion (*M*)	−0.073	0.048	[−0.166, 0.021]	0.126
Dispositional mindfulness (*W*)	−0.187	0.038	[−0.263, −0.112]	<0.001
*X* × *W*	−0.018	0.034	[−0.084, 0.049]	0.603
*R* ^2^	0.123			
Panel C. Problematic mealtime behaviors equation				
Primary caregiver	−0.186	0.072	[−0.328, −0.045]	0.010
Child sex	−0.070	0.063	[−0.194, 0.054]	0.267
Child age	−0.048	0.029	[−0.104, 0.008]	0.095
Only-child status	0.057	0.077	[−0.095, 0.208]	0.465
Household registration	0.057	0.077	[−0.094, 0.208]	0.459
Household annual income	0.024	0.049	[−0.072, 0.120]	0.625
Parental phubbing (*X*)	0.245	0.064	[0.119, 0.371]	<0.001
Family cohesion (*M*)	−0.165	0.055	[−0.272, −0.057]	0.003
Dispositional mindfulness (*W*)	−0.135	0.045	[−0.224, −0.047]	0.003
*X* × *W*	0.007	0.052	[−0.095, 0.109]	0.894
*R* ^2^	0.138			

Note. *N* = 550. Coefficients are unstandardized. HC3 = heteroskedasticity–consistent standard error. Parental phubbing and parental dispositional mindfulness were mean-centered before the interaction term was formed. Coding: primary caregiver, 0 = father, 1 = mother; child sex, 0 = boy, 1 = girl; only–child status, 0 = only child, 1 = non–only child; household registration, 0 = rural, 1 = urban; household annual income, 0 = 0–100,000 CNY, 1 = 100,001–500,000 CNY, 2 = above 500,000 CNY.

## Data Availability

The data that support the findings of this study are available on request from the corresponding author. The data are not publicly available due to their containing information that could compromise the privacy of research participants.

## Appendix A

**Table A1:** Targeted Confirmatory Factor Analysis Model Comparisons.

Construct Comparison	Model	*χ* ^2^	*df*	CFI	TLI	RMSEA [90% CI]	SRMR
Eating outcomes	Two-factor	244.98 ***	53	0.873	0.842	0.081 [0.071, 0.092]	0.073
	One-factor	503.07 ***	54	0.704	0.638	0.123 [0.113, 0.133]	0.097
Phubbing vs. dispositional mindfulness	Two-factor	893.67 ***	251	0.888	0.876	0.068 [0.063, 0.073]	0.051
	One-factor	1726.72 ***	252	0.742	0.717	0.103 [0.099, 0.108]	0.095

Note. *** *p* < 0.001. CFI = comparative fit index; TLI = Tucker–Lewis index; RMSEA = root mean square error of approximation; SRMR = standardized root mean square residual.

**Table A2:** Measurement Quality and Standardized Factor Loadings.

Construct/Item	Standardized Loading	CR	AVE	Latent *r*	HTMT
Panel A. Eating outcomes					
Picky eating		0.745	0.309		
E1	0.700				
E2	0.746				
E3	0.539				
E4	0.404				
E5	0.609				
E6	0.370				
E7	0.394				
Problematic mealtime behaviors		0.758	0.386	0.526	0.598
E8	0.530				
E9	0.616				
E10	0.643				
E11	0.643				
E12	0.667				
Panel B. Phubbing and dispositional mindfulness					
Parental phubbing		0.818	0.367		
A1	0.539				
A2	0.544				
A3	0.682				
A4	0.644				
A5	0.698				
A6	0.714				
A7	−0.050				
A8	0.574				
A9	0.715				
Parental dispositional mindfulness	B1–B15: 0.491–0.794	0.922	0.445	−0.533	0.558

Note. CR = composite reliability; AVE = average variance extracted; HTMT = heterotrait–monotrait ratio. Latent *r* and HTMT are shown on the second construct in each paired comparison.

**Table A3:** Full Item List for Picky Eating and Problematic Mealtime Behaviors.

Item	Subscale	Chinese Wording	English Translation
E1	Picky eating	我的小孩只吃自己选择的那些食物。	My child only eats foods that they choose.
E2	Picky eating	我的小孩因为气味、口味、外观、质地的原因拒绝很多食物。	My child refuses many foods because of their smell, taste, appearance, or texture.
E3	Picky eating	我的孩子不吃以前没有吃过的食物。	My child does not eat foods that they have not eaten before.
E4	Picky eating	吃饭时，大人给什么我的小孩就吃什么 *。	At meals, my child eats whatever adults give them. (Reverse-worded).
E5	Picky eating	我的小孩会把不想吃的食物扔掉或吐出来。	My child throws away or spits out food that they do not want to eat.
E6	Picky eating	我的孩子喜欢多种食物 *。	My child likes a variety of foods. (Reverse-worded).
E7	Picky eating	我的小孩常常因为饭菜发脾气。	My child often loses their temper because of the food served.
E8	Problematic mealtime behaviors	我的小孩能乖乖地坐下吃完一顿饭 *。	My child can sit properly and finish a meal. (Reverse-worded).
E9	Problematic mealtime behaviors	我的小孩在吃饭时把饭菜含在嘴里很长时间不咽。	During meals, my child keeps food in their mouth for a long time without swallowing.
E10	Problematic mealtime behaviors	我的孩子吃饭时间长，超过半小时。	My child takes a long time to eat, exceeding 30 min.
E11	Problematic mealtime behaviors	我的小孩吃饭时要看电视、玩玩具或讲故事。	My child needs to watch television, play with toys, or listen to stories while eating.
E12	Problematic mealtime behaviors	我的小孩在正餐前吃零食或点心，而在正餐时不好好吃东西。	My child eats snacks before regular meals and then does not eat properly at mealtime.

Note. Chinese wording is reproduced from the questionnaire administered in the study; the English column provides corresponding translations. An asterisk (*) marks items that were reverse-worded in the original questionnaire. Reverse-worded items had been recoded before composite scores were calculated.

**Table A4:** Formal Between-Outcome Comparisons in the Primary Joint Model.

Comparison (Problematic Mealtime Behaviors−Picky Eating)	Difference	*SE*/Boot *SE*	95% CI	*p*
Direct phubbing effect at low mindfulness	0.158	0.056	[0.047, 0.268]	0.005
Direct phubbing effect at mean mindfulness	0.177	0.055	[0.070, 0.284]	0.001
Direct phubbing effect at high mindfulness	0.197	0.080	[0.040, 0.354]	0.014
Family cohesion *b*-path	−0.092	0.053	[−0.196, 0.012]	0.082
Direct *X* × *W* path	0.025	0.054	[−0.081, 0.130]	0.646
Conditional indirect association at low mindfulness	0.001	0.005	[−0.011, 0.013]	0.886
Conditional indirect association at mean mindfulness	0.012	0.008	[−0.001, 0.030]	0.089
Conditional indirect association at high mindfulness	0.023	0.014	[−0.003, 0.052]	0.084
Index of moderated mediation	0.014	0.009	[−0.002, 0.032]	0.084

Note. Direct-path and *b*-path comparisons use cross-equation HC3 robust covariance estimates. Conditional indirect and index comparisons use 5000 case-resampling bootstrap samples. The *p* values are unadjusted; Holm-adjusted values are reported in Table A7.

**Table A5:** Conditional Indirect Associations and Indices of Moderated Mediation.

Outcome/Moderator Level	Effect	Boot *SE*	95% Bootstrap CI	Bootstrap *p*
Panel A. Picky eating				
Low mindfulness (−1 *SD*)	0.001	0.004	[−0.009, 0.010]	0.910
Mean mindfulness	0.009	0.007	[−0.002, 0.025]	0.125
High mindfulness (+1 *SD*)	0.018	0.013	[−0.005, 0.045]	0.120
Index of moderated mediation	0.011	0.008	[−0.003, 0.028]	0.120
Panel B. Problematic mealtime behaviors				
Low mindfulness (−1 *SD*)	0.001	0.009	[−0.017, 0.018]	0.880
Mean mindfulness	0.021	0.010	[0.004, 0.044]	0.008
High mindfulness (+1 *SD*)	0.041	0.016	[0.013, 0.075]	0.002
Index of moderated mediation	0.025	0.010	[0.008, 0.048]	0.002

Note. Bootstrap *p* values are empirical two-sided values based on 5000 case-resampling bootstrap samples. Conditional effects are evaluated at −1 *SD*, the mean, and +1 *SD* of parental dispositional mindfulness.

**Table A6:** Supplementary Joint Simple Mediation Model.

Predictor/Effect	*b*/*ab*	HC3 *SE*/Boot *SE*	95% CI	*p*/Boot *p*
Panel A. Family cohesion equation				
Parental phubbing	−0.253	0.041	[−0.333, −0.173]	<0.001
Panel B. Picky eating equation				
Parental phubbing	0.168	0.042	[0.085, 0.251]	<0.001
Family cohesion	−0.144	0.046	[−0.234, −0.053]	0.002
Indirect association (*X* → *M* → *Y*)	0.036	0.013	[0.013, 0.063]	0.003
Panel C. Problematic mealtime behaviors equation				
Parental phubbing	0.310	0.058	[0.197, 0.423]	<0.001
Family cohesion	−0.219	0.054	[−0.325, −0.112]	<0.001
Indirect association (*X* → *M* → *Y*)	0.055	0.015	[0.027, 0.086]	<0.001
Panel D. Between-outcome comparisons				
Direct phubbing path difference	0.142	0.050	[0.044, 0.240]	0.005
Family cohesion *b*-path difference	−0.075	0.050	[−0.173, 0.023]	0.135
Indirect association difference	0.019	0.013	[−0.006, 0.045]	0.134

Note. All regression equations included the same six covariates as the primary model. Indirect associations and their differences use 5000 case–resampling bootstrap samples. Regression-path *p* values use HC3 robust standard errors.

**Table A7:** Simple-Slope and Johnson–Neyman Probes of the Phubbing × Dispositional Mindfulness Interaction.

Probe	Estimate/Range	HC3 *SE*	95% CI	*p*/Sample Proportion
Simple slope at low mindfulness (−1 *SD*)	−0.008	0.052	[−0.109, 0.092]	0.870
Simple slope at mean mindfulness	−0.130	0.046	[−0.219, −0.040]	0.004
Simple slope at high mindfulness (+1 *SD*)	−0.251	0.056	[−0.360, −0.141]	<0.001
Johnson–Neyman region: *W* < 2.71	Slope statistically different from zero			6/550 (1.1%)
Johnson–Neyman region: 2.71 ≤ *W* ≤ 4.28	Slope not statistically different from zero			191/550 (34.7%)
Johnson–Neyman region: *W* > 4.28	Negative slope statistically different from zero			353/550 (64.2%)

Note. *W* = raw parental dispositional mindfulness score. Johnson–Neyman transition values are approximately 2.71 and 4.28. The lower significant region contained very few observations and was not substantively interpreted.

**Table A8:** Holm-Adjusted Tests for Multiplicity.

Test	Raw *p*	Holm-Adjusted *p*
Panel A. Prespecified hypothesis-level tests		
H1a: Phubbing-picky eating correlation	<0.001	<0.001
H1b: Phubbing-problematic mealtime correlation	<0.001	<0.001
H2a: Simple indirect association, picky eating	0.003	0.011
H2b: Simple indirect association, problematic mealtime behaviors	<0.001	0.002
H3: *X* × *W* → family cohesion	<0.001	<0.001
H4a: *X* × *W* → picky eating	0.603	1.000
H4b: *X* × *W* → problematic mealtime behaviors	0.894	1.000
H5a: Index of moderated mediation, picky eating	0.120	0.359
H5b: Index of moderated mediation, problematic mealtime behaviors	0.002	0.008
Panel B. Formal between-outcome comparison family		
Direct phubbing path	0.001	0.008
Family cohesion *b*-path	0.082	0.489
Difference in moderated mediation indices	0.084	0.489
Conditional indirect difference, high *W*	0.084	0.489
Conditional indirect difference, mean *W*	0.089	0.489
Direct *X* × *W* path	0.646	1.000
Conditional indirect difference, low *W*	0.886	1.000

Note. Holm corrections were applied separately within the two test families. Bootstrap-based hypothesis tests used empirical two-sided bootstrap *p* values. Follow-up simple slopes and Johnson–Neyman probes were not treated as separate confirmatory tests.
